# Primary Surgical Therapy for Locally Limited Oral Tongue Cancer

**DOI:** 10.1155/2014/738716

**Published:** 2014-05-20

**Authors:** Konstantinos Mantsopoulos, Georgios Psychogios, Julian Künzel, Frank Waldfahrer, Johannes Zenk, Heinrich Iro

**Affiliations:** Department of Otorhinolaryngology, Head and Neck Surgery, University of Erlangen-Nuremberg, Waldstraße 1, 91054 Erlangen, Germany

## Abstract

*Objectives*. The aim of this study was to assess the efficacy of primary surgical treatment in the management of locally limited oral tongue carcinoma. *Methods*. A retrospective evaluation was carried out for all patients treated with primary surgery for pT1-pT2 oral tongue carcinomas at a tertiary referral center between 1980 and 2005. All cases were assessed for disease-specific survival and local control rates in relation to T classification, N classification, infiltration depth of the primary tumor, and decision making on neck management and adjuvant therapy. The cases were additionally evaluated for the incidence of major complications and tracheotomies. *Results*. 263 cases were assessed. The 5-year disease-specific survival rate was 75.2%. Positive neck disease was shown to be a significant negative prognostic factor. The occult metastasis rate was 20.2%. *Conclusions*. Primary surgical treatment is a very effective modality against T1-T2 oral tongue carcinoma, and a low rate of complications can be anticipated.

## 1. Introduction

The oral tongue is the subsite most frequently affected by carcinomas in the oral cavity [1]. It is well known that malignant lesions in this region have a strong propensity to develop neck metastases, [2] which are observed even in the early stages of the disease [3].

The management of a cN0 neck in patients with advanced (T3-T4) primary tumors is not an issue, as flap reconstruction and thus neck exploration are required in most cases. The tumors are irradiated in most cases, and the neck can be irradiated together with the oral cavity [2]. However, whether neck management is mandatory (with selective neck dissection or irradiation) in patients with T1-T2 lesions continues to be a matter of debate [1]. Patients with occult metastases may benefit from management of the neck, but the question has not yet been conclusively settled [1, 4].

The purpose of the present study was to evaluate experience in the primary surgical management of local early oral tongue squamous cell carcinomas at a single oncology referral center for head and neck cancers. An additional aim was to obtain information about the regional metastatic behavior of these carcinomas and the different factors influencing it, as this is relevant to decision making regarding neck management. Finally, an analysis of complications and the rate of tracheotomies needed was performed.

## 2. Materials and Methods

A retrospective study was conducted at an academic tertiary referral center (the Department of Otorhinolaryngology, Head and Neck Surgery, University of Erlangen-Nuremberg, Erlangen, Germany). The files for all patients treated with primary surgery for early (T1 and T2) local oral tongue carcinomas between 1980 and 2005 were evaluated. Patients, who had previously undergone treatment for the same reason and had systemic disease at the time of diagnosis, histological findings other than squamous cell carcinoma, or second primary tumors at the time of diagnosis, were excluded from the study.

All pathology reports were reviewed, and staging was conducted in accordance with the 2010 American Joint Committee on Cancer (AJCC) and Union Internationale Contre le Cancer (UICC) classification [5]. In the TNM staging system, T1 lesions are defined as those in which the largest diameter is 2 cm or less, whereas T2 lesions are larger than 2 cm but not more than 4 cm in their largest diameter. Approval was obtained from the institutional review board of the hospital.

All of the cases were assessed for 5-year overall survival (OS), 5-year disease-specific survival (DSS), and local control (LC) rates in relation to the T classification, N classification, decision on adjuvant therapy, and depth of tumor infiltration. Adjuvant treatment in this series consisted of postoperative radiotherapy (interstitial or percutaneous), either alone or combined with chemotherapy. Typical indications for adjuvant treatment included the presence of positive surgical margins when further surgery was not feasible, advanced neck disease, a tumor infiltration depth of more than 5 mm, extracapsular tumor spread, and infiltration of lymph vessels or nerves on histology. The decision of whether to offer adjuvant therapy was also affected by the choice of surgical management of the neck. Percutaneous irradiation typically included the primary tumor site and both sides of the neck. Interstitial brachytherapy included only the primary tumor site.

OS was estimated from the time of diagnosis to the time of death. DSS was defined using the time from the date of diagnosis to death from the cancer or complications of treatment. The time to LC was calculated from the date of the initial diagnosis to the date of the most recent clinical review at which local recurrence was confirmed. Local recurrence was defined as invasive carcinoma developing at the anatomic site of the primary tumor after completion of the initial treatment. Statistical analysis was performed using the Kaplan-Meier method with 95% confidence intervals. SPSS version 19 for Windows (SPSS Inc., Chicago, IL, USA) was used for statistical analysis. A *P* value of <0.05 was considered statistically significant.

Major complications were defined as those that required prolonged hospitalization, blood transfusion, additional surgery, or admission to the intensive-care unit. Tracheotomies were considered to be transient when performed intraoperatively or in the immediate postoperative period but later closed. They were considered permanent in cases in which surgical closure was not possible at any time postoperatively.

## 3. Results

A total of 263 patients were finally included in the study. Among these, 188 were men and 75 women, with a male-female ratio of 2.51 : 1. Their mean age was 57.7 years (range: 31–92 years; SD 11.6). The mean follow-up period was 73.3 months (Table 1). The overall survival (OS) was 56.9%, the DSS was 75.2%, and the LC rate was 86.3% in this series. According to the pathology reports, negative surgical margins (R0 status) were achieved in 260 patients (98.8%). Three patients (1.2%) had positive surgical margins (R+ status) at the end of surgical treatment. These patients refused further surgery. Transoral resection of the tumor was performed in 243 cases (conventional 213, laser 30) and a transoral-transcervical approach was used in 20 cases (Table 1).

**Figure 1:**
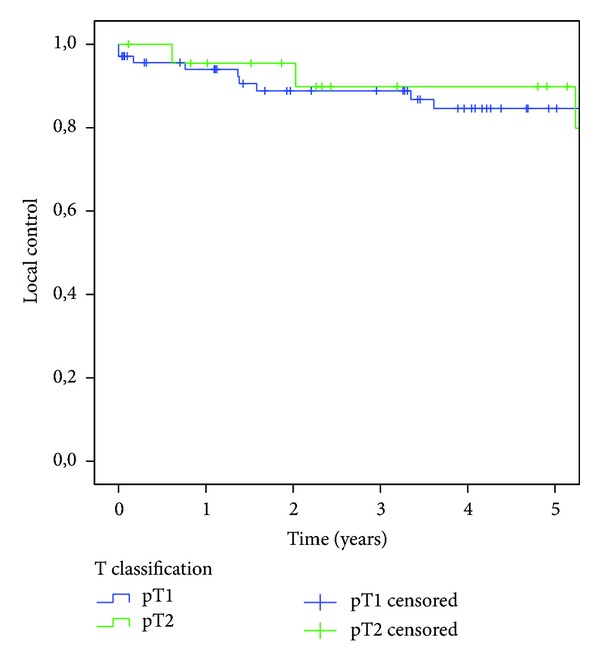
Kaplan-Meier analysis of disease-specific survival relative to T status in the group of N0 patients without adjuvant therapy.

A total of 132 patients had pT1 oral tongue tumors (50.2%), while 131 (49.8%) had pT2 tumors. Considering only the group of N0 patients without adjuvant therapy, we found 93 cases (70 T1 and 23 T2 tumors) without any significant difference in the LC rates (81.5 versus 89.8% resp.;   *P* = 0.874) (Figure 1). OS was 70.1% for patients with T1 oral tongue cancer and 40.4% for those with T2 tumors (*P* = 0.4). The DSS was 89.6% in the T1 group and 69.3% in the T2 group (*P* = 0.189). The LC rates were 83.5% and 81.8%, respectively (*P* = 0.41).

**Figure 2:**
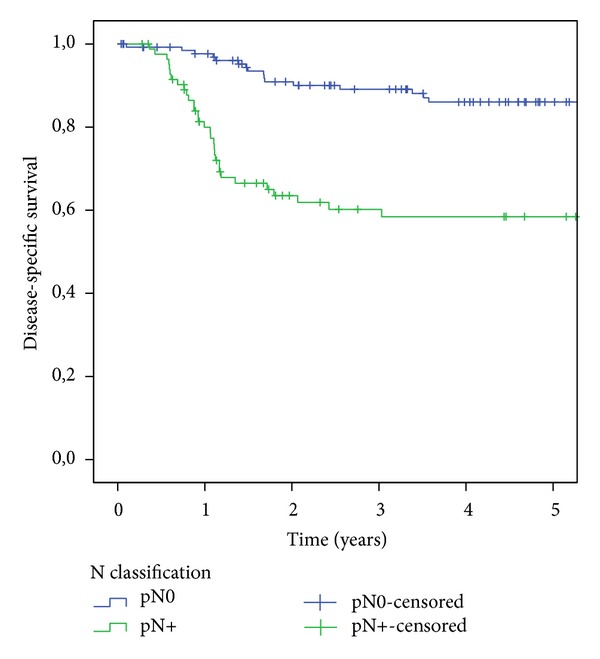
Kaplan-Meier analysis of disease-specific survival relative to N status.

From the 263 patients of our study, 118 were cN0 and 145 were cN+. In the cN0 group, OS was 66.7% and DSS was 81.6%. In the cN+ group, OS was 49.4% and DSS was 70.2%. Positive lymph nodes (pN+) were found in 85 of the 263 patients (32.3%). If only the 218 cases with neck dissection are taken into account, the metastasis rate was higher (38.9%). In the subgroup of these 218 cases OS was 64.9% for patients with pN0 status and 43.4% for those with pN+ status (*P* = 0.000). The DSS was 86.0% in the pN0 group and 58.4% in the pN+ group (*P* = 0.000) (Figure 2).

Neck dissection was performed in 79 of the 118 patients with cN0 status (in 70 cases unilaterally and in 9 cases on both sides). Of these 79 patients, 16 had pN+ status (eight with pN1, one with pN2a, and seven with pN2b). The rate of occult metastasis was thus 20.2%. In the group with occult metastases, OS and DSS were 68.2%. No significant influence of the T classification on the rate of occult metastases was noted (*P* = 0.617). Occult metastases on the contralateral neck side were found in one of the 9 patients receiving a bilateral neck dissection (11.1%).

Six patients had metastases on the contralateral neck side (two with pT1 tumors and four with pT2 tumors). An analysis of these patients' characteristics showed that five of the six (83.3%) had cN2c status preoperatively, while all of them (100%) already had metastases on the ipsilateral neck side. Five of them (83.3%) also had infiltration of the midline of the oral tongue; only one, thus, had a strictly contralateral metastasis.

Exclusively surgical treatment was carried out in 109 of the patients (41.4%), and 154 patients (58.6%) received combined therapy (primary surgery and adjuvant percutaneous radiotherapy/brachytherapy in 136 cases; primary surgery and chemoradiotherapy in 18 cases). The OS rate was 55.4% in the first group and 57.8% for those with combined therapy. The DSS rates were 74.8% and 75.4%, respectively. The LC rates were 82.3% and 89%, respectively (*P* = 0.147).

46 patients (17.5%) required reoperation (twice in 45 patients; three times in one case). Patients with one operation in the primary site had an OS of 61.7%, while the rate in those with more operations was 47.9% (*P* = 0.072). The DSS was 81.3% in the first group and 60.5% in the second (*P* = 0.008). The LC rates were 89.2% in the first group and 76.4% in the second (0.079).

**Figure 3:**
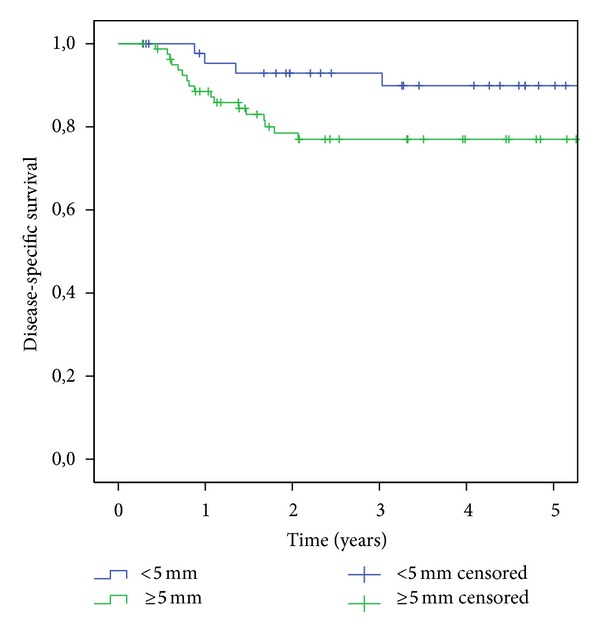
Kaplan-Meier analysis of disease-specific survival relative to infiltration depth.

Patients with an infiltration depth of less than 5 mm (68/263, 25.9%) had an OS of 70.8%, while the rate in those with an infiltration depth ≥5 mm (195/263, 74.1%) was 48.2% (*P* = 0.013). The DSS was 90.3% in the first group and 76.5% in the second (*P* = 0.03; Figure 3). The LC rates were 96.3% in the first group and 87.3% in the second (*P* = 0.108). In total, 16 patients (10 T2 and 6 T1) with an infiltration depth of 5 mm or less and cN0 status underwent neck dissection. In this group, three (2/10 T2 and 1/6 T1) had occult metastases (in total 18.8%). Of the 63 patients (43 T2 and 20 T1) with an infiltration depth of more than 5 mm and cN0 status who underwent neck dissection, 13 (20.6%) had occult metastases (9/43 T2 and 4/20 T1). This means that the incidence of occult metastases in the T1 group was 16.7% by an infiltration depth <5 mm and 20% over 5 mm. The incidence rates of positive nodal disease in the different patient groups relative to the infiltration depth are shown in Table 2. No statistically significant differences were observed between the different groups with regard to regional metastatic behavior.

Major complications in this series included bleeding, aspiration, fistula formation, wound healing problems, and nerve injury. None of these complications was fatal. A detailed presentation is given in Table 3. The overall incidence of complications was 7.6%. Permanent tracheotomies were necessary in two cases (0.7%) and temporary tracheostomies in four cases overall (1.4%).

## 4. Discussion

A review of the relevant literature shows that early local oral tongue carcinomas present a clinical dilemma, due to the lack of prospective randomized trials [6]. To the best of our knowledge, the case series of surgically managed early local oral tongue carcinomas presented here is the largest in the literature to date. The relatively high rates of disease-specific survival (75.2%) and local control (86.3%) in the analysis, together with the low complication rate (7.6%) and the extremely low number of tracheotomies, support the hypothesis that surgery can provide an effective and safe cure for these cases without significant compromises in relation to the posttherapeutic quality of life.

Neck dissection was performed in 218 of the 263 patients in the study sample (82.9%). Forty-five patients (17.1%) did not receive surgical neck treatment, either because they declined therapy or because they had comorbid medical problems. According to the literature, positive nodal disease is one of the most influential prognostic factors in patients with head and neck carcinomas [7–9] This was confirmed by the present analysis: positive neck disease (pN+) was detected in 34.2% of our cases and was associated with a poorer prognosis, affecting the overall and disease-specific survival rates to a highly significant extent. Interestingly, according to the present analysis, a patient suffering from a small carcinoma of the oral tongue with regional metastases is 4.2 times more likely to die of the disease during the year following the completion of therapy in comparison with a patient with N0 status. This emphasizes the crucial need for management of the cervical lymph nodes with neck dissection or irradiation, even in patients with local early carcinomas.

The management of the neck in patients with cN0 status continues to be a matter of controversy [4, 10]. In general, the “principle of 15–20%” [4, 11, 12] (referring to the incidence of occult metastases) represents an acceptable compromise between the morbidity of elective neck dissection and the need for salvage treatment later on [13]. According to the relevant literature, the rate of occult metastases in patients with early local oral tongue carcinomas can be as high as 42% [14, 15]. An overall occult metastasis rate of 20.2% was noted in the present study, higher than the “threshold” mentioned above, and thus generally suggesting a need for elective neck management (in the form of neck dissection or irradiation) in all early local oral tongue carcinomas patients with cN0 status, in accordance with similar studies [16].

Our analysis showed that the majority of early local oral tongue carcinomas (97.3%) were strictly lateral lesions, with no involvement of the tip or midline of the tongue. Six of the 263 cases (2.3%) had metastases on the other side, and this was not influenced by the size of the primary tumor. All six patients already had suspect nodes preoperatively on the same side, all but one with infiltration of the midline. It might therefore be argued that management of the contralateral neck side in patients with early local oral tongue carcinomas could possibly be reserved for clinical N2c status or for lesions with infiltration of the tip or midline of the tongue.

Franceschi et al. suggested that treatment of the primary tumor site and ipsilateral elective neck management in patients with lingual carcinomas may predispose toward tumor cell migration to the opposite side of the neck [17]. Only sparse literature reports on this topic have been published to date. Lim et al. reported an occult nodal disease rate of 4% on the contralateral side, with no benefit from contralateral elective neck dissection in comparison with “watchful waiting” [16]. In the present series, occult metastases on the contralateral neck side were found in only one case (1.7%). If only the cases with bilateral neck dissection are examined, the occult contralateral metastases rate remains remarkably low (11.1%). Adequate examination of the neck region (with palpation and imaging studies) might possibly be able to reduce the incidence of occult neck disease on the contralateral side. Patient compliance should always be taken into consideration, of course, and regular follow-up is essential.

In an effort to detect subgroups in the present study in which it might be possible to avoid ipsilateral neck management (the associated morbidity of which is not negligible), the incidence of occult nodal disease was investigated in relation to different primary tumor characteristics. A smaller primary tumor size (T1) was not associated with a significantly lower rate of occult metastases. Furthermore, almost 19% of the patients with an infiltration depth of 5 mm or less already had subclinical nodal disease. The number of T1 with an infiltration depth of 5 mm or less who underwent a neck dissection was extremely low in our study sample, so that no safe conclusions as to avoidance of neck management in this subgroup can be drawn. It is though remarkable that even the smallest primary tumors of our study sample had an occult metastasis rate of almost 17%! These data indicate the aggressiveness of the disease at this primary site and suggest that elective neck dissection cannot be avoided—particularly in view of the value of neck management as a staging procedure [8, 9].

With regard to the oncologic parameters of the study, no statistically significant differences were found between patients with and without adjuvant therapy. The absence of differences might be due to the fact that patients who received combined therapy usually had poorer prognostic characteristics, such as advanced neck disease or a tumor infiltration depth of more than 5 mm. Carrying out adjuvant therapy may therefore have contributed to eliminating possibly significant differences due to these negative prognostic factors. It should be emphasized that a lack of homogeneity with regard to different protocols of adjuvant treatment may be regarded as a limitation of the present analysis.

Interestingly, our analysis showed that patients with R0-situation after more than one surgical procedure on the primary site had significantly poorer oncologic parameters in comparison with “single-operation” patients. These data point to the fact that a subsequent reoperation, if dictated by permanent histology, will carry a negative effect on local disease control and survival of the patients. It is, therefore, highly recommended to take this aspect into consideration and always try to achieve R0-situation in local early oral tongue carcinomas by means of one operation.

Among head and neck cancers, the oral cavity is the location in which tumor parameters such as infiltration depth and tumor thickness have been most studied [6]. Some authors measure it from the deepest point of invasion to the most protruding tumor surface [18], whereas others define it as the distance from the deepest tumor invasion point to an imaginary line along the adjacent healthy mucosa [19, 20]. In the present authors' view, using a virtual line along the adjacent healthy mucosa and thus measuring only the endophytic part of the lesion could provide a more precise estimate of the aggressiveness of the tumor, as has also been proposed by Gonzalez-Moles et al. [6] With tumor infiltration depth defined as described above, it was found that a cut-off level of 5 mm is decisive for the course of the disease, with significantly poorer OS and DSS and a trend toward more frequent local recurrence in more deeply infiltrating carcinomas. The latter might possibly be explained by the difficulty of assessing the deep tumor margin accurately with palpation during tumor resection, potentially with a greater likelihood of inadequate resection margins in the deep layers of surgical dissection [21]. The gradual increase in the rate of regional metastases in deeper primary lesions might be explained by the fact that microinvasion or contraction of the lingual musculature promotes the entry of cancer cells into the lymphatics [21]. Even with superficial lesions (<5 mm), our analysis showed a remarkably high overall positive node rate (29.4%, Table 2) and a not negligible rate of occult metastases (18.8%), so that a primary aggressive approach toward neck management appears to be justified in all cases, irrespective of the infiltration depth. The analysis thus does not support the approach of waiting for the pathology report on the tumor specimen before a decision is made on neck management. The considerable advantages of treating the primary site and the regional lymphatic system in one surgical procedure (with less morbidity) should be emphasized [21]. According to our study results, preoperative estimation of the tumor infiltration depth (e.g., through palpation or imaging studies) does not appear to be crucial for the planning of neck management and could be possibly used only to assess the prognosis. This approach may, of course, involve a risk of overtreatment in some patients, [14] and individualization of treatment (ensuring a high level of patient compliance and regular follow-up) is therefore a reasonable and not suboptimal approach.

## 5. Conclusions

Critical analysis of our data supported the hypothesis that surgery can provide an effective and safe cure for these cases without significant compromises in relation to the posttherapeutic quality of life. Positive neck disease was associated with a significantly poorer prognosis. A noted overall occult metastasis rate of 20.2% generally suggested a need for elective neck management (in the form of neck dissection or irradiation) in cN0 cases. Furthermore, R0-situation after more than one surgical procedure was shown to have significantly poorer oncologic parameters in comparison with “single-operation” patients. Interestingly, it was found that a cut-off level of 5 mm is decisive for the course of the disease, with significantly poorer survival rates and a trend toward more frequent local recurrence in more deeply infiltrating carcinomas. Nevertheless, a primary aggressive approach toward neck management appears to be justified in all cases, irrespective of the infiltration depth.

## Figures and Tables

**Table 1:** Demographic and tumor-specific characteristics of the study patients.

Characteristics	Results
Age (years)	Mean: 57.7, median: 56, and range: 31 to 92
Follow-up (months)	Mean: 73.3, range: 24–300
Gender	Male: 188 (71.5), female: 75 (28.5)
Surgical technique (%)	Transoral laser microsurgery: 30 (11.4), electrocautery: 213 (81), and combined transoral-transcervical approach: 20 (7.6)
Adjuvant treatment (%)	No adjuvant therapy: 109 (41.4), RT: 136 (51.7), and RCT: 18 (6.8)
PT classification (%)	pT1: 132 (50.2), pT2: 131 (49.8)
PN classification (%)	pN0: 133 (50.6), pN1: 25 (9.5), pN2: 48 (18.3), pN3: 12 (4.6), and no neck dissection: 45 (17.1)
Differentiation	Well: 66 (25), moderate: 155 (58.9), poor: 39 (14.8), and not differentiated: 3 (1.1)

**Table 2:** Incidence rate of positive nodal disease relative to the infiltration depth of small carcinomas of the mobile tongue.

Infiltration depth	Number of patients (%) with overall positive nodal disease by infiltration depth
<2 mm	3/15 (20)
<3 mm	8/30 (26.6)
<4 mm	16/54 (29.6)
<5 mm	20/68 (29.4)
<6 mm	28/92 (30.4)
<7 mm	32/106 (30.2)
<8 mm	42/128 (32.8)

**Table 3:** Specific types and incidence of surgical complications.

Complication	Number of complications (%)
Bleeding	10 (3.8)
Aspiration	2 (0.7)
Fistula	2 (0.7)
Wound healing disorders	1 (0.7)
Nerve lesions	3 (1.1)
Other complications	2 (0.7)

## References

[1] Veness MJ, Morgan GJ, Sathiyaseelan Y, Gebski V (2005). Anterior tongue cancer and the incidence of cervical lymph node metastases with increasing tumour thickness: should elective treatment to the neck be standard practice in all patients?. *ANZ Journal of Surgery*.

[2] Yuen APW, Lam KY, Lam LK (2002). Prognostic factors of clinically stage I and II oral tongue carcinoma—a comparative study of stage, thickness, shape, growth pattern, invasive front malignancy grading, Martinez-Gimeno score, and pathologic features. *Head and Neck*.

[3] Yuen APW, Lam KY, Chan ACL (1999). Clinicopathological analysis of elective neck dissection for no neck of early oral tongue carcinoma. *American Journal of Surgery*.

[4] Psychogios G, Mantsopoulos K, Bohr C, Koch M, Zenk J, Iro H (2013). Incidence of occult cervical metastasis in head and neck carcinomas: development over time. *Journal of Surgical Oncology*.

[5] Gospodarowitz M, Wittekind C, Sobin L (2009). AJCC cancer staging manual. *TNM Classification of Malignant Tumours*.

[6] Gonzalez-Moles MA, Esteban F, Rodriguez-Archilla A, Ruiz-Avila I, Gonzalez-Moles S (2002). Importance of tumour thickness measurement in prognosis of tongue cancer. *Oral Oncology*.

[7] Teichgraeber JF, Clairmont AA (1984). The incidence of occult metastases for cancer of the oral tongue and floor of the mouth: treatment rationale. *Head and Neck Surgery*.

[8] Mantsopoulos K, Psychogios G, Waldfahrer F, Zenk J, Iro H (2012). Surgical treatment of locally limited tonsillar cancer. *Surgical Oncology*.

[9] Mantsopoulos K, Psychogios G, Bohr C (2012). Primary surgical treatment of T3 glottic carcinoma: long-term results and decision-making aspects. *The Laryngoscope*.

[10] Psychogios G, Mantsopoulos K, Koch M (2013). Elective neck dissection vs observation in transorally treated early head and neck carcinomas with cN0 neck. *Acta Oto-Laryngologica*.

[11] Weiss MH, Harrison LB, Isaacs RS (1994). Use of decision analysis in planning a management strategy for the stage NO neck. *Archives of Otolaryngology—Head and Neck Surgery*.

[12] Pitman KT (2000). Rationale for elective neck dissection. *American Journal of Otolaryngology*.

[13] Sparano A, Weinstein G, Chalian A, Yodul M, Weber R (2004). Multivariate predictors of occult neck metastasis in early oral tongue cancer. *Otolaryngology—Head and Neck Surgery*.

[14] O-charoenrat P, Pillai G, Patel S (2003). Tumour thickness predicts cervical nodal metastases and survival in early oral tongue cancer. *Oral Oncology*.

[15] Ho CM, Lam KH, Wei WI, Lau SK, Lam LK (1992). Occult lymph node metastasis in small oral tongue cancers. *Head and Neck*.

[16] Lim YC, Lee JS, Koo BS, Kim S-H, Kim Y-H, Choi EC (2006). Treatment of contralateral N0 neck in early squamous cell carcinoma of the oral tongue: elective neck dissection versus observation. *Laryngoscope*.

[17] Franceschi D, Gupta R, Spiro RH, Shah JP (1993). Improved survival in the treatment of squamous carcinoma of the oral tongue. *American Journal of Surgery*.

[18] Brown B, Barnes L, Mazariegos J, Taylor F, Johnson J, Wagner RL (1989). Prognostic factors in mobile tongue and floor of mouth carcinoma. *Cancer*.

[19] Myers JN, Elkins T, Roberts D, Byers RM (2000). Squamous cell carcinoma of the tongue in young adults: increasing incidence and factors that predict treatment outcomes. *Otolaryngology—Head and Neck Surgery*.

[20] Asakage T, Yokose T, Mukai K (1998). Tumor thickness predicts cervical metastasis in patients with stage I/II carcinoma of the tongue. *Cancer*.

[21] Yuen APW, Lam KY, Wei WI (2000). A comparison of the prognostic significance of tumor diameter, length, width, thickness, area, volume, and clinicopathological features of oral tongue carcinoma. *American Journal of Surgery*.

